# Sexual dysfunction after surgery for primary sporadic cranial meningiomas: prevalence and risk factors

**DOI:** 10.1007/s11060-024-04817-w

**Published:** 2024-09-10

**Authors:** Alim Emre Basaran, Felix Arlt, Erdem Güresir, Martin Vychopen, Johannes Wach

**Affiliations:** 1 Department of Neurosurgery, University Hospital Leipzig, Liebigstraße 20, 04103 Leipzig, Germany; 2 Comprehensive Cancer Center Central Germany, Partner Site Leipzig, Leipzig, Germany

**Keywords:** Cranial meningioma, Sexual dysfunction, Quality of life

## Abstract

**Background:**

Although postoperative quality of life (QoL) has been studied in relation to a variety of aspects following meningioma resection, the impact of meningiomas on sexual life has not been investigated. The aim of this study is to determine the impact of cranial meningioma surgery on patients’ postoperative sexual life.

**Methods:**

A standardized questionnaire, anonymous and based on the Arizona Sexual Experiences Scale (ASEX), was sent to 87 patients who had been selected for participation in the study based on the following criteria: a postoperative Karnofsky performance of ≥ 80 and below 60 years of age at diagnosis.

**Results:**

53 patients (53/87; 61%) responded to the survey. The study identified eleven patients (20.8%) who reported sexual dysfunction (SD) according to ASEX criteria. Six of these patients were women (55%) and five were men (45%). Univariable analysis revealed that SD was observed with greater frequency in patients with non-skull base tumors (*p* = 0.006) and in those with a left-hemispheric meningioma (*p* = 0.046). Multivariable analysis revealed that non-skull base tumor location is the only independent factor being associated with SD (OR = 5.71, 95% CI = 1.02–31.81, *p* = 0.047).

**Conclusion:**

This first investigation of sexual functioning post-surgery for cranial meningiomas indicates that SD is a prevalent issue among non-skull base meningioma patients. Consequently, we recommend that pre- and postoperative sexual health should be further addressed in future QoL investigations of cranial meningioma patients.

**Graphical Abstract:**

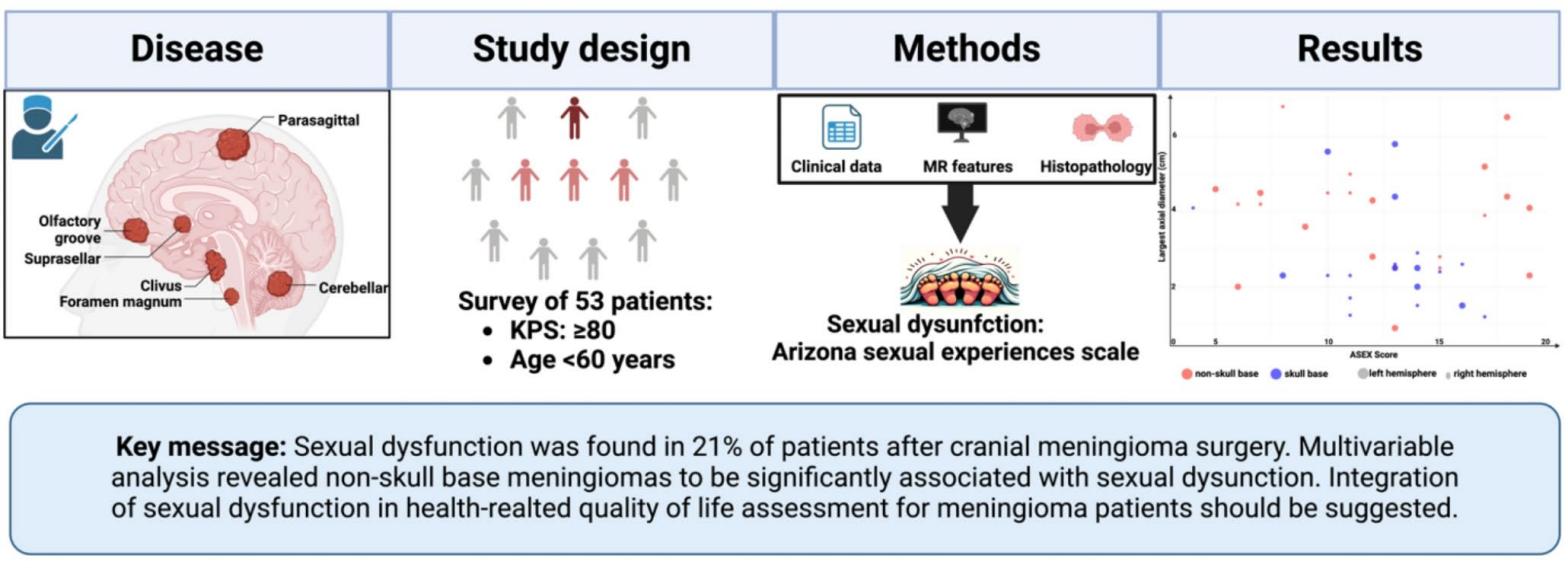

## Introduction

The sexual health of cancer patients is of great importance to their overall well-being and quality of life (QoL). Sexual dysfunction (SD) can lead to a number of accompanying psychological and social disorders, significantly impairing the QoL of those affected [1]. SDs in gynecological and urological diseases that affect sexual life are the subject of intensive research and extensive findings [2–4]. *Sexual dysfunction is a recognized issue in patients undergoing brain tumor surgery. While the impact of intra-axial tumors like gliomas on neural networks and subsequent sexual dysfunction has been studied*,* there is a lack of research on the effects of extra-axial tumors such as meningiomas on sexual function* [5].

Meningiomas can affect individuals of all age groups, with the highest incidence observed in between the fifth and sixth decade of age [6]. Rates of perioperative complications are higher in elderly patients, which implicates that surgical treatment if deemed necessary, should be performed in earlier stages of life [7]. However, the wish for child and sexual functioning is an essential part in the preoperative decision-making process and the lifespan of younger meningioma patients. Owens et al. revealed that 70% of young female patients diagnosed with meningioma expressed a desire to have children [8]. In contrast, earlier diagnosis because of improved MR-imaging modalities, the implementation of more effective surgical techniques and targeted stereotactic radiotherapy, both progression-free survival and overall survival of meningioma patients have improved significantly in recent years [9]. Given the paucity of efficacious therapeutic options for meningiomas and the superior surgical techniques currently available, surgical treatment is the treatment of choice in the majority of cases [10].

Nevertheless, it is imperative that aggressive surgical resection of the tumor with respect of a preserved good QoL is of importance. Despite the extensive research conducted on postoperative QoL in meningioma patients with neurological and neurocognitive deficits, no study on SD after cranial meningioma surgery only has been published to date [11, 12]. Our study is the first dedicated to this topic. This study is the first to evaluate SD in a homogeneous group of primary sporadic cranial meningioma patients who have undergone surgery and maintain a good postoperative physical functioning.

## Materials and methods

The patients were selected from the institutional consecutive meningioma database who had undergone surgery between 2011 and 2021. IRB approval was obtained from the local ethic committee (No:165/24-ck). Given the investigation of sexual functioning and the potential topic of child wish, we limited our study to younger meningioma patients under the age of 60 and with Karnofsky performance status (KPS) ≥ 80 at discharge. The additional inclusion criteria were as follows: In order to be eligible for inclusion in the study, patients had to be between 18 and 60 years of age at time of surgery, have no postoperative neurological deficit, no previous malignancies and long-term diseases, no previous radiotherapy or previous chemotherapy, and be able to return to a normal professional and social environment. Skull-base tumors include olfactory groove, planum ethmoidale-sphenoidale, parasellar, tuberculum sellae, clival-petroclival, and foramen magnum meningiomas, lateral and middle sphenoid wing meningiomas, temporal fossa, spheno-orbital meningiomas, as well as meningiomas of the petrous bone and occipital fossa. Non-skkull base tumors include convexity, parasagittal, falx, tentorium, cerebellar convexity, pineal region, and intraventricular meningiomas [13]. The meningioma location, peritumoral edema, tumor volume, and tumor surface area were determined by pre- and postoperative MRI images using Gd-enhanced T1-weighted sequences with 3D Slicer (Version 5.2.1, Surgical Planning Laboratory, Harvard University, USA). Extent of resection was graded according to the Simpson classification system [14]. Histopathological grading of the present cohort was performed according to the 2016 WHO criteria [15]. Neuropathological findings of the patients prior to the 2016 WHO grading system underwent a review regarding the exclusion of brain invasion. Immunohistochemistry including determination of the MIB-1 labeling index was conducted in a similar way as previously reported for paraffin-embedded biopsy tissue specimen [16].

### Questionnaire

An online survey was designed via the online platform Google Forms (www.google.com/forms/about). The structure of the survey regarding sexual functioning was designed as previously described for low-grade gliomas [5]. The survey was voluntary and anonymous, and it was available from January 2024 to April 2024. The questionnaire was created in the German language. The distribution process began with contact by telephone and an introductory email that provided a brief overview of the survey’s objectives and purposes. It is important to highlight that participants were not offered any financial compensation for completing the survey. The survey contained statements on the following subjective aspects regarding sexual health: participants were asked to indicate whether their current sexual life had improved, worsened or remained unchanged compared to their preoperative state. The Arizona Sexual Experiences Scale (ASEX) is a validated, concise, 5-item rating scale created to evaluate the primary aspects of sexual function: sex drive, arousal, penile erection/vaginal lubrication, ability to reach orgasm, and orgasmic satisfaction. Each question had an answer option with a 6-point scale, with higher scores indicating greater impairment. An ASEX total score of ≥ 19, any single item scored at ≥ 5, or any three items scored at ≥ 4 are all associated with sexual dysfunction [17].

### Statistics

Recorded data were imported into a computational database in SPSS version 29.0 (IBM, Armonk, NY, USA). Categorical data and continuous data were compared using Fisher’s exact test (two-sided) and t-tests or Mann-Whitney U tests, respectively. Only variables being significant (*p*-value < 0.05) in the univariable analysis were included in the multivariable binary logistic regression analysis of factors being potentially associated with SD. Statistical graphics were created using R version 4.3.1 (R Foundation for Statistical Computing, Vienna, Austria). The forest plot and bubble plot charts were created using the R package ggplot2.

## Results

Of the 150 patients contacted, 83 (55%) were willing to participate in the study. The questionnaire was then sent electronically to the 83 patients. Of these 83 patients, 53 (63.9%) completed the entire questionnaire.

### Patient characteristics

The cohort comprised 53 patients with cranial meningiomas. The mean age at diagnosis was 47.0 years (SD ± 8.5). The majority of the patients were female (73.6%, *n* = 39) while males constituted 26.4% (*n* = 14) of the cohort. Regarding tumor location, 43.4% (*n* = 23) of the tumors were non-skull base, whereas 56.6% (*n* = 30) were skull base. Peritumoral edema was present in 41.5% (*n* = 22) of the patients. The mean tumor volume was 19.0 cm³ (SD ± 20.6). Tumors were located on the left side in 41.5% (*n* = 22) of the patients, on the right side in 50.9% (*n* = 27), and in the midline in 7.5% (*n* = 4). Simpson grade distribution was as follows: 54.7% (*n* = 29) grade 1, 30.2% (*n* = 16) grade 2, 0% (*n* = 0) grade 3, and 15.1% (*n* = 8) grade 4. WHO grade distribution was predominantly grade 1 (92.5%, *n* = 49), with grade 2 in 5.7% (*n* = 3) and grade 3 in 1.8% (*n* = 1). The mean time since surgery was 84.0 months (SD ± 44.6). Further characteristics are summarized in Table 1.

**Table 1:** Patient characteristic

Age at diagnosis (mean ± SD)	47.0 +/-8.5
Sex	
Female	39 (73.6%)
Male	14 (26.4%)
Location	
Non-skull base	23 (43.4%)
Skull base	30 (56.6%)
Peritumoral edema	
Present	22 (41.5%)
Absent	31 (58.5%)
Tumor volume (mean ± SD, cm³)	19.0 ± 20.6
Tumor surface area (mean ± SD, cm²)	50.8 ± 76.5
Tumor side	
Left	22 (41.5%)
Right	27 (50.9%)
Midline	4 (7.5%)
Simpson grade	
1	29 (54.7%)
2	16 (30.2%)
3	0 (0.0%)
4	8 (15.1%)
WHO grade	
1	49 (92.5%)
2	3 (5.7%)
3	1 (1.8%)
MIB-1 labeling index (mean ± SD)	4.8 ± 5.3
Time since surgery (mean ± SD, months)	84.0 ± 44.6
Adjuvant radiotherapy	
Received	1 (1.9%)
Not received	52 (98.1%)
Postoperative simple partial seizures	
Yes	5 (9.4%)
No	48 (90.6%)

### Univariate analysis of sexual dysfunction

Seventeen out of 53 patients (32%) reported a subjective change in their sexual life, of which 14 (82%) reported a worsening and 3 (18%) an improvement.

In the present cohort of 53 cranial meningioma patients, 11 (20.8%) fulfilled criteria for SD, while 42 (79.2%) did not fulfill the criteria for diagnosis of SD according to ASEX. Univariable analysis revealed that tumor location and tumor side are associated with SD after cranial meningioma surgery. Patients without SD were more likely to have skull base tumors (93.3%) compared to those with SD (6.7%) (*p* = 0.006). Non-skull base tumors were more prevalent in patients with SD (39.1%) compared to those without (60.9%). Tumor side also showed a significant correlation with SD: 63.6% of left-sided tumors were in patients without SD versus 36.4% with SD, and 88.9% of right-sided tumors were in the no SD group compared to 11.1% with SD (*p* = 0.046, excluding midline tumors).

No significant differences were found regarding sex, age at diagnosis, tumor size, tumor volume, tumor surface area histopathology, extent of resection, and adjuvant treatment. Mean tumor volumes were 18.7 ± 21.0 cm³ for patients without sexual dysfunction and 20.3 ± 20.0 cm³ for those with it (*p* = 0.83). WHO grades and the MIB-1 labeling index showed no significant differences (*p* = 0.57 and *p* = 0.48, respectively). Surgical resection (Simpson grade) and adjuvant radiation therapy did not differ significantly between groups (*p* = 0.81 and *p* = 0.99, respectively). The incidence of postoperative simple partial seizures was also not significantly different (*p* = 0.28). Table 2 summarizes the univariable results.

**Table 2:** Patient-, disease- and treatment-specific characteristics of cranial meningioma patients with or without sexual dysfunction

Characteristic	No sexual dysfunction (42/53; 79.2%)	Sexual dysfunction (11/53; 20.8%)	*p*-value
Age at diagnosis	46.2 +/- 8.7	49.8 +/- 7.1	0.21
Sex			0.13
Female	33 (84.6%)	6 (15.4%)	
Male	9 (64.3%)	5 (35.7%)	
Diabetes mellitus			0.51
Present	2 (4.8%)	1 (9.1%)	
Absent	40 (95.2%)	10 (90.9%)	
NSAID intake			0.99
Present	3 (7.1%)	0 (0.0%)	
Absent	39 (92.9%)	11 (100%)	
Antihypertensive			0.73
Medication			
Present	15 (35.7%)	5 (45.5%)	
Absent	27 (64.3%)	6 (54.5%)	
Location			0.006
Non-skull base	14 (60.9%)	9 (39.1%)	
Skull base	28 (93.3%)	2 (6.7%)	
Calcification			0.99
Present	2 (4.8%)	0 (0.0%)	
Absent	40 (95.2%)	11 (100	
Peritumoral edema	18 (81.8%)	4 (18.2%)	0.75
Largest axial diameter, cm, mean +/- SD	3.3 cm +/- 1.5	3.5 +/- 1.4	0.66
Tumor volume (cm^3^), mean +/- SD	18.7 +/- 21.0 cm^3^	20.3 +/- 20.0 cm^3^	0.83
Tumor surface area (cm^2^), mean +/- SD	54.4 +/- 83.3 cm^2^	37.0 +/- 26.0 cm^2^	0.52
Tumor side			0.046 (excluding midline tumors)
Left	14 (63.6%)	8 (36.4%)	
Right	24 (88.9%)	3 (11.1%)	
Midline	4 (100%)	0 (0%)	
Simpson grade			0.81
1	23 (54.8%)	6 (54.6%)	
2	12 (28.6%)	4 (36.4%)	
3 4	0 (0.0%) 7 (16.7%)	0 (0.0%) 1 (9.1%)	
WHO grade 1	38 (77.6%)	11 (22.4%)	0.57
2	3 (100%)	0 (0%)	
3	1 (100%)	0 (0%)	
MIB-1 labeling index, mean +/- SD	5.1 +/- 5.8	3.8 +/- 2.2	0.48
Time since surgery (months), mean +/- SD	81.1 +/- 41.1	94.6 +/- 54.3	0.37
Adjuvant radiation therapy	1 (2.4%)	0 (0.0%)	0.99
Postoperative simple partial seizures	3 (60.0%)	2 (40.0%)	0.28

The individual data and distribution of answers of each individual participant among the five items of the ASEX scale are summarized in Table 3.

**Table 3:** Patient specific responses on ASEX questionnaire

Patient	Subjective change	Tumor Side	Location	ASEX	SD	Q1	Q2	Q3	Q4	Q5
1	worsened	right hemisphere	medial sphenoid wing	4	no	2	1	0	0	1
2	worsened	right hemisphere	convexity	6	no	1	1	1	1	2
3	worsened	right hemisphere	medial sphenoid wing	6	no	1	2	NA	1	2
4	worsened	right hemisphere	convexity	7	yes	0	2	0	0	5
5	worsened	right hemisphere	convexity	8	no	1	1	2	2	2
6	unchanged	right hemisphere	parasagittal	10	no	2	2	2	2	2
7	worsened	right hemisphere	medial sphenoid wing	10	yes	1	1	5	1	2
8	unchanged	right hemisphere	lateral sphenoid wing	11	no	1	2	3	3	2
9	unchanged	right hemisphere	medial sphenoid wing	11	no	2	2	2	2	3
10	unchanged	right hemisphere	convexity	11	no	2	2	2	2	3
11	unchanged	right hemisphere	convexity	11	no	1	2	3	3	2
12	unchanged	right hemisphere	medial sphenoid wing	11	no	2	2	2	2	3
13	unchanged	right hemisphere	lateral sphenoid wing	12	no	3	2	3	2	2
14	unchanged	right hemisphere	spheno-orbital	13	no	2	3	0	4	4
15	unchanged	right hemisphere	convexity	13	no	1	3	3	3	3
16	unchanged	right hemisphere	spheno-orbital	13	no	1	2	4	2	2
17	unchanged	right hemisphere	spheno-orbital	14	no	2	2	3	3	4
18	unchanged	right hemisphere	parasellar	14	no	2	3	4	3	4
19	unchanged	right hemisphere	convexity	15	no	3	2	3	2	2
20	unchanged	right hemisphere	tentorium	15	yes	2	3	4	3	4
21	unchanged	right hemisphere	petroclival	15	no	2	3	4	3	2
22	unchanged	right hemisphere	medial sphenoid wing	15	no	2	3	3	3	4
23	unchanged	right hemisphere	medial sphenoid wing	16	no	3	3	3	3	4
24	unchanged	right hemisphere	convexity	17	no	3	3	3	3	4
25	unchanged	right hemisphere	petroclival	17	no	3	3	4	3	4
26	worsened	left hemisphere	parasagittal	5	yes	1	1	2	1	3
27	worsened	left hemisphere	falx	6	yes	2	2	3	2	4
28	worsened	left hemisphere	tentorium	7	no	2	3	2	2	2
29	worsened	left hemisphere	parasellar	8	yes	3	3	2	2	2
30	worsened	left hemisphere	convexity	9	yes	2	2	3	3	2
31	unchanged	left hemisphere	medial sphenoid wing	10	no	2	2	2	3	4
32	unchanged	left hemisphere	parasagittal	12	no	3	2	3	3	3
33	unchanged	left hemisphere	falx	12	no	3	2	4	2	2
34	unchanged	left hemisphere	falx	13	no	2	3	2	2	2
35	unchanged	left hemisphere	petroclival	13	no	3	3	2	2	2
36	unchanged	left hemisphere	lateral sphenoid wing	13	no	2	3	2	2	2
37	unchanged	left hemisphere	lateral sphenoid wing	13	no	1	3	3	3	3
38	unchanged	left hemisphere	parasellar	14	no	3	3	4	2	2
39	unchanged	left hemisphere	medial sphenoid wing	14	no	2	3	1	4	4
40	unchanged	left hemisphere	medial sphenoid wing	16	no	3	3	3	3	4
41	unchanged	left hemisphere	convexity	17	no	3	4	3	3	4
42	unchanged	left hemisphere	falx	17	no	3	3	3	4	4
43	unchanged	left hemisphere	falx	18	yes	3	3	4	4	4
44	unchanged	left hemisphere	convexity	18	yes	3	4	4	4	3
45	improved	left hemisphere	parasagittal	19	yes	3	4	4	4	4
46	improved	left hemisphere	falx	19	yes	3	4	4	4	4
47	unchanged	both sides	olfactory groove	12	no	3	3	2	2	2
48	unchanged	both sides	olfactory groove	13	no	3	3	2	3	2
49	improved	both sides	olfactory groove	15	no	2	3	3	3	4
50	worsened	both sides	tuberculum sellae	15	no	2	3	3	3	2
51	unchanged	both sides	tuberculum sellae	16	no	3	3	3	3	4
52	worsened	both sides	medial sphenoid wing	17	no	3	3	3	4	4
53	worsened	both sides	medial sphenoid wing	17	no	3	3	3	4	4

**Fig. 1:**
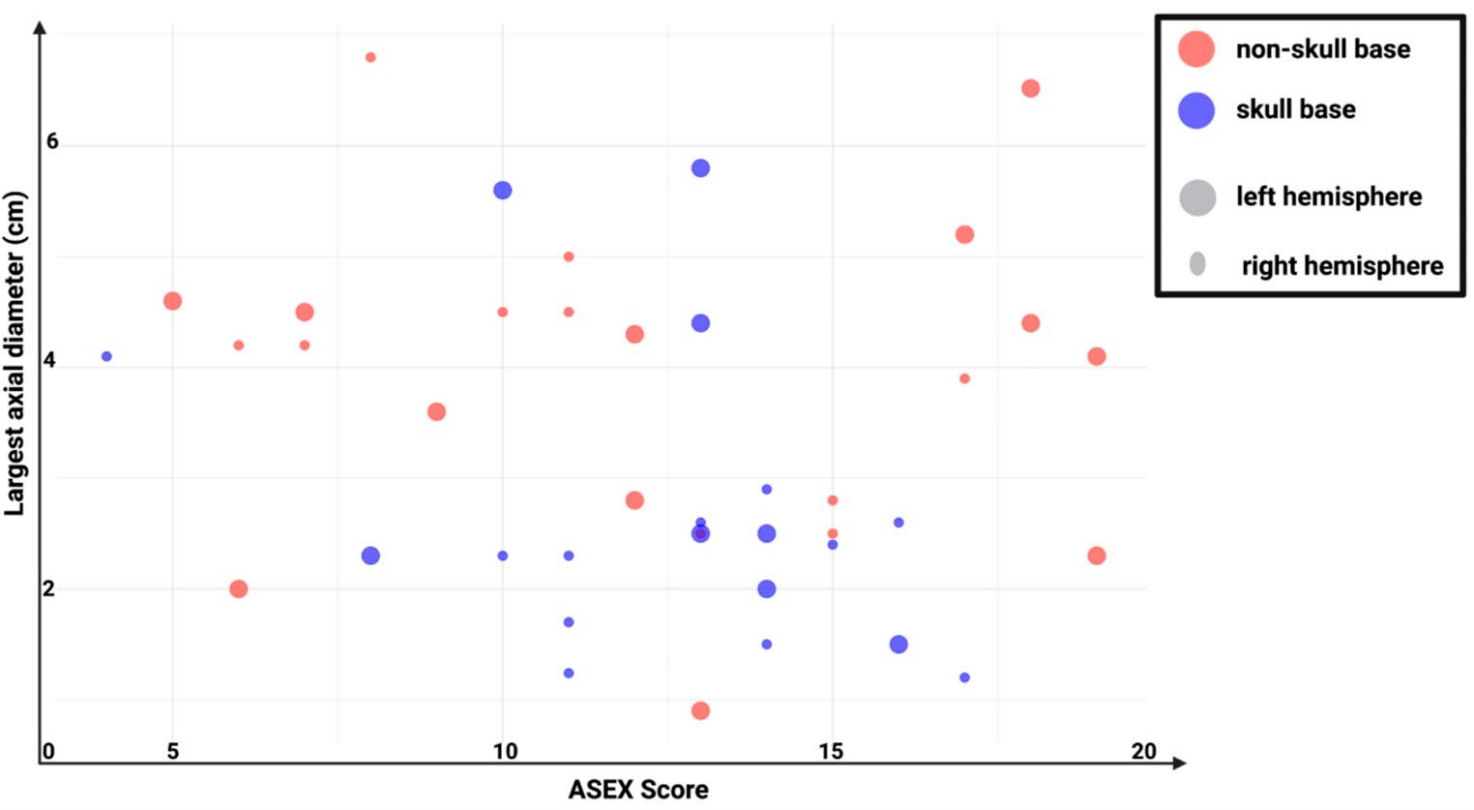
Bubble plot of ASEX Score versus largest axial meningioma diameter (in cm) in both non-skull base and skull-base meningiomas. The plot compares ASEX scores with the largest axial diameter of tumors, with data points color-coded by tumor location: non-skull base (red) and skull base (blue). Data points are also stratified to indicate the hemisphere: Larger bubbles constitute the left hemisphere and smaller bubbles constitute right hemisphere. Midline meningiomas were excluded in this illustration

The neuroanatomical location of brain tumors is of crucial importance for perioperative management and postoperative QoL. Of the participants, 23 (43%) had a non-skull base tumor and 30 (57%) had a skull base tumor. The results from univariable analysis suggest that patients with a left-sided non-skull base tumors have higher ASEX scores and significantly worse sexual life as classified by the diagnostic criteria for SD according to ASEX responses compared to those with skull base tumors Fig. 1.

### Multivariable analysis of sexual dysfunction after cranial meningioma surgery

**Fig. 2:**
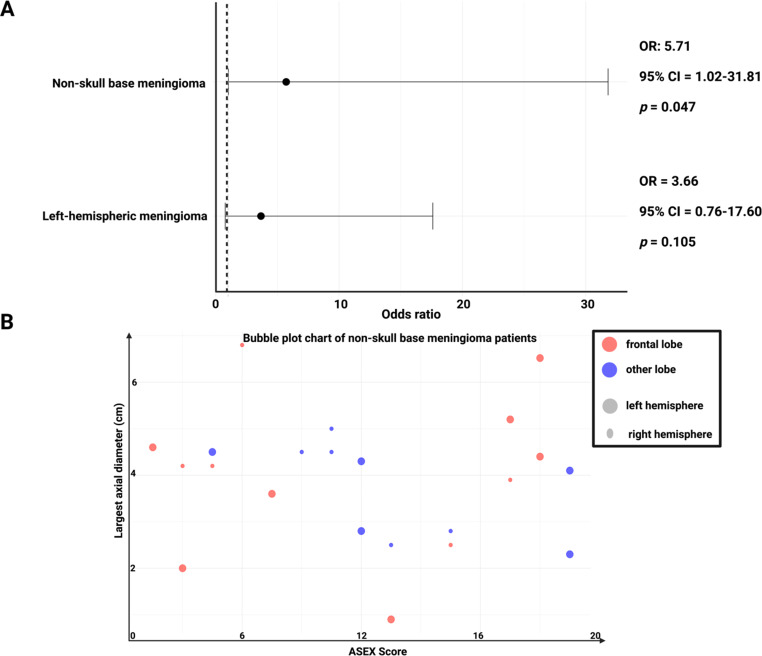
(**A**) Forest plot of factors associated with sexual dysfunction after cranial meningioma surgery. The plot presents odds ratios (OR) with 95% confidence intervals (CI) from a multivariable binary logistic regression analysis. Non-skull base meningiomas have an OR of 5.71 (95% CI: 1.02–31.81, *p* = 0.047), indicating a significant association with increased risk of sexual dysfunction. The dashed vertical line represents a reference line for an odds ratio of 1.00. (**B**) Bubble plot of ASEX Score versus largest axial meningioma diameter (in cm) in non-skull base meningiomas. The plot compares ASEX scores with the largest axial diameter of tumors, with data points color-coded by tumor lobe anatomy: frontal (red) and non-frontal (blue). Data points are also stratified to indicate the hemisphere: Larger bubbles constitute the left hemisphere and smaller bubbles constitute right hemisphere

Multivariable binary logistic regression analysis of factors being associated with SD was performed with the consideration of the following univariate significant parameters: Tumor location (non-skull base vs. skull base) and tumor side (left hemispheric vs. right hemispheric). In multivariable logistic regression, non-skull base tumor location remained significantly associated with sexual dysfunction (OR = 5.71, 95% CI = 1.02–31.81, *p* = 0.047), while the association with tumor side was not statistically significant (OR = 3.67, 95% CI = 0.76–17.60, *p* = 0.11, see Fig. 2.

### Subgroup review of non-skull base meningiomas

We performed a further analysis of the 23 non-skull base meningioma patients. Seven (77.8%) out of 9 non-skull base meningiomas with a SD had a frontal non-skull base meningioma, whereas 6 (42.9%) of those 14 non-skull base meningioma patients without SD had a frontally located tumor (Fisher’s exact test (2-sided): *p* = 0.20). As far as laterality of the non-skull base meningiomas is concerned, 777.8%) out of 9 non-skull base meningiomas with a SD had a left-hemispheric non-skull base meningioma, whereas 6 (42.9%) of those 14 non-skull base meningioma patients without SD had a right-hemispheric tumor (Fisher’s exact test (2-sided): *p* = 0.20). Figure 2B visualizes the non-skull base meningioma patients in a bubble plot chart.

## Discussion

This study demonstrates that SD in patients who have undergone sporadic cranial primary meningioma surgery is a prevalent issue that is not uncommon among this neuro-oncological disease. In their study, Boccia et al. demonstrated a correlation between brain tumors and sexual dysfunction. However, the study population was highly heterogeneous with different histopathological brain tumor entities (e.g., low-grade gliomas, high-grade gliomas, meningiomas, acoustic neuroma, medulloblastoma) comprising only 12 meningioma patients, only two of whom were men [18]. Given the limited number of cases, it is not possible to draw a definitive conclusion regarding the relationship between meningioma and SD. To date, there is no known literature that has investigated SD following cranial meningioma surgery. This is also due to the sensitive and taboo subject of sexuality, as both healthy people and cancer patients might be reluctant to discuss their sexual experiences and dysfunctions [19]. It is also important to note that WHO grade 1 meningiomas are benign long-term chronic disease due their potential to cause symptoms and the need for repeated neurosurgical intervention and neuro-oncological follow-up [11, 20]. The current questionnaires used to monitor QoL, such as EQ-5D [21], SF-36 [22], PROMIS [23], FACT-BR [24], MDASI-BT [25], and BN20 [26], do not include patient-reported outcome measures regarding sexual functioning during or after cancer therapy. While the QLQ-C30 [27] and QLQ-BN20 [28] contain a single question with the wording “I am satisfied with my sex life,” no conclusion can be drawn from this one question.

The absence of a neuro-oncological specific sexuality-related questionnaire makes the 5-point ASEX rating scale an optimal instrument for assessing and evaluating sexuality. Indeed, 21% of our patients exhibited postoperative SD according to the ASEX scale. Demographic parameters such as age and sex were comparable among those with or without SD. Previous studies on postoperative sexuality have been limited to epilepsy surgery, with a particular focus on temporal lobe epilepsy surgery and the regulatory role of the amygdala [29]. The only study investigating postoperative deterioration in sexual functioning after brain tumor surgery was the study by Surbeck et al., who retrospectively analyzed sexuality in 32 low-grade glioma patients [5]. They found that 44% of the low-grade glioma patients fulfilled the criteria of SD. In the present study of 53 cranial sporadic meningiomas 21% fulfilled the criteria of SD, respectively. Hence, our study indicates that SD is also common after cranial meningioma surgery and potentially worsens general well-being and postoperative QoL. Due to the intimate nature of the questionnaire, it can be assumed that the response rate with 63.9% was low, and that the proportion of SD might be even higher. Against this backdrop, it can be argued that a high proportion exists due to the psychological pressure caused by brain tumor disease.

In contrast to gynecological and urological diseases such as breast cancer, ovarian cancer, uterine cancer and prostate cancer [30], SD was considered to have a low prevalence among meningioma patients. However, this was not confirmed in our study. This finding is of importance because cancer patients with SD exhibited higher levels of anxiety and depression, which are associated with a poorer QoL [31]. Furthermore, SD might be also a result from cancer-related fatigue [32]. Despite evidence from several studies on sexual functioning in terms of central nervous system processes in the literature, there is still considerable controversy with regard to the results in relation to SD and the role of the cerebral hemispheres [33, 34]. The present study was able to demonstrate that patients who have undergone surgery for non-skull base meningiomas are statistically more likely to suffer from SD. Studies indicate that a disrupted neuronal structure in the insula, amygdala, and orbitofrontal cortex can also result in SD. This corroborates our hypothesis that SD can occur in non-skull-base meningiomas. One of most common sites for meningiomas are the frontal lobe, which plays a key role in regulating sexual behavior and function. Furthermore, the majority of the non-skull base meningiomas in the present series were located in the frontal region. The frontal lobe, particularly the orbitofrontal cortex, is integral in the cognitive and emotional processes underlying sexual behavior. Disruptions in this area can lead to alterations in sexual desire, arousal, and overall sexual function [35]. The association between meningiomas and the frontal brain, coupled with the frontal brain’s critical role in sexuality, provides strong evidence for the relevance of our hypothesis. The potentially disrupted neuronal structures in the insula, amygdala, and orbitofrontal cortex seen in meningioma patients highlight the broader impact these tumors can have on sexual health and behavior.

Previous knowledge of neurophysiology and neuroanatomy in relation to sexual sensations and sexual auras is based on patients suffering from epileptic seizures. Sexual arousal and orgasmic ecstasy were found to be measured by stimulation of the mesiotemporal lobe and basal forebrain structures [36, 37]. Consequently, further studies are required to ascertain the impact of resection sites on sexual functioning after cranial meningioma surgery. Furthermore, future studies will have to correlate their findings with established QoL questionnaires.

### Limitations

The major limitation of the present investigations were the potential biases from nonresponse and self-selection, given the voluntary design of the present study. Hence, the current survey is limited by nonresponse bias, in that those who were not willing or not able to participate might be different from those who participated. Additionally, the multifactorial nature of SD represents a significant source of potential bias, as it complicates the attribution of SD to a single cause and introduces the possibility of bias. Furthermore, the patient-, disease-, and treatment-specific characteristics were retrospectively recorded. Nevertheless, we applied highly selective inclusion criteria (age < 60, KPS ≥ 80, sporadic tumors) to ensure that only young patients with good physical functioning and potentially good QoL were included. Our results suggest that multiple potential factors influence sexual dysfunction rather than confirming a direct causal relationship between surgery and sexual dysfunction. It would be beneficial for future studies to use a prospective longitudinal design with preoperative assessments of sexual function and longer follow-up periods to better differentiate the contributions of the tumor, surgery, and other confounding factors to postoperative sexual dysfunction.

## Conclusion

Although the topic has not been addressed in the literature, our study demonstrates that SD following meningioma surgery is a prevalent issue among non-skull base meningiomas. Given that many doctors are not well-versed in the topic of sexual health and patients are reluctant to inquire due to the sensitivity of the subject matter, it is of paramount importance to explicitly address this topic and offer patients medical assistance and support when necessary. Given the improved therapeutic options, associated improved recurrence, and overall survival, it is critical to better understand and predict the impact of these surgeries to prevent SD and improve QoL. Future large-scale investigations of non-skull base meningioma patients regarding this endpoint are needed.

## Data Availability

No datasets were generated or analysed during the current study.
